# Clinical characteristics of 41 patients with pneumonia due to 2019 novel coronavirus disease (COVID-19) in Jilin, China

**DOI:** 10.1186/s12879-020-05677-1

**Published:** 2020-12-17

**Authors:** Qing Zhang, Qian Xu, Yi-yang Chen, Li-xin Lou, Li-he Che, Xiao-hua Li, Lu-yao Sun, Wan-guo Bao, Na Du

**Affiliations:** 1 Infectious Diseases Department, First Hospital of Jilin University, Changchun, 130021 Jilin China; 2 Integrated Chinese and Western Medicine Department, the Infectious Diseases Hospital of Changchun City, Changchun, 130123 Jilin China

**Keywords:** COVID-19, Clinical characteristics, ACE2, Jilin, cTnI

## Abstract

**Background:**

The clinical characteristics of patients with confirmed 2019 novel coronavirus disease (COVID-19) in Jilin Province, China were investigated.

**Methods:**

Clinical, laboratory, radiology, and treatment data of 41 hospitalized patients with confirmed COVID-19 were retrospectively collected. The population was stratified by disease severity as mild, moderate, or severe, based on guidelines of the National Health and Medical Commission of China.

**Results:**

The 41 hospitalized patients with COVID-19 were studied, and the median age was 45 years (interquartile range [IQR], 31–53; range, 10–87 years) and 18 patients (43.9%) were female. All of the patients had recently visited Wuhan or other places (ie, Beijing, Thailand) or had Wuhan-related exposure. Common symptoms included fever (32[78%]) and cough (29[70.7%]). All patients were without hepatitis B/C virus hepatitis. CRP (C-reactive protein, 11.3 mg/L [interquartile range {IQR}, 2.45–35.2]) was elevated in 22 patients (53.7%), and cardiac troponin I (1.5 ng/mL [IQR, 0.8–5.0]) was elevated in 41 patients (100%). Chest computed tomographic scans showed bilateral ground glass opacity (GGO) or GGO with consolidation in the lungs of 27(65.9%) patients. 31(75.6%) patients had an abnormal electrocardiograph (ECG). Comparing the three groups, the levels of CRP and cardiac troponin I, GGO distribution in bilateral lungs, and electrocardiogram changes were statistically significant (*p* < 0.05). Cardiac troponin I had a strong positive correlation with CRP (r = 0.704, *p* = 0.042) and LDH (r = 0.738, *p* = 0.037).

**Conclusion:**

Significant differences among the groups suggest that several clinical parameters may serve as biomarkers of COVID-19 severity at hospital admission. Elevated cTnI could be considered as a predictor of severe COVID-19, reflecting the prognosis of patients with severe COVID-19. The results warrant further inspection and confirmation.

## Introduction

Beginning in December 2019, a series of unexplained cases of viral pneumonia occurred in Wuhan City, Hubei province. During the following 2 months, the disease broke out in other part of China and internationally as well [1–4]. It was a major public health emergency in China, with the fastest spread, over a wider geographic range, and the most difficult to control, since the founding of New China.

The Coronavirus Research Group of the International Virus Classification Committee changed the name of the causative virus 2019-nCoV to severe acute respiratory syndrome coronavirus 2 (SARS-CoV-2), which was subsequently named by the WHO as coronavirus disease 2019 (COVID-19).

Coronaviruses include SARS-CoV (severe acute respiratory syndrome coronavirus) and MERS-CoV (Middle East respiratory syndrome coronavirus). SARS-CoV-2 is the seventh identified species of human coronavirus, and is most similar to SARS-CoV (77.2% nucleotide similarity) [2, 5–11]. SARS-CoV-2 infections in humans, with person-to-person transmission, probably began at a seafood market in Wuhan [2, 3, 5–8, 12, 13]. Wang et al. [8] mentioned that common symptoms include fever, fatigue and dry cough. Low lymphocyte count and elevated LDH in the laboratory and bilateral lungs with GGO in lung CT are the typical features in Wuhan.

Herein, we report early clinical, laboratory, and radiologic features of 41 patients with confirmed diagnosis of SARS-CoV-2 infection in Changchun, Jilin Province. The clinical features of patients with mild, moderate, and severe disease were compared [14].

## Methods

This retrospective study was approved by the Ethics Committee of First Hospital of Jilin University and Infectious Diseases Hospital in Changchun City, Jilin Province (No.2020–399). The need to obtain informed consent from the patients was waived.

### Patients

The local government of Changchun City, Jilin Province mandated the treatment of patients infected with COVID-19 to First Hospital of Jilin University and Infectious Disease Hospital. This study enrolled all patients with COVID-19 who were admitted to these hospitals from 23 January 2020 to 25 February 2020. The diagnoses of COVID-19, inclusion and exclusion criteria, and criteria for hospital admission were in accordance with the guidelines of the National Health Commission of China.

### Data collection

The clinical information, laboratory results, and chest computed tomography (CT) features of all the patients were collected from the electronic medical network of the hospitals. To ensure accuracy of the data, two independent researchers reviewed and checked the data forms.

### Stratification of patients by severity of disease

All patients were classified as having mild (ie, nonpneumonia), moderate (ie, pneumonia), or severe disease (ie, dyspnea, respiratory frequency 30/min, blood oxygen saturation 93%, partial pressure of arterial oxygen to fraction of inspired oxygen ratio 50% within 24 to 48 h), based on the novel Coronavirus-Infected Pneumonia Diagnosis and Treatment Plan (Trial Version 7) announced by the National Health and Medical Commission of China [14].

### Statistical analysis

Continuous variables are reported as means and standard deviations or medians and interquartile ranges. Categorical variables are shown as number and percentage, and comparisons of the mild, the moderate, and severe groups were performed with the Fisher’s exact test. When comparing multiple groups, the Kruskal-Wallis H test is required for continuous variables. After the test, a pairwise comparison is required. In order to make the calculation result more accurate and reduce Type I errors, Bonferroni correction must be performed. And the correlation coefficient was derived Pearson’s correlation analysis. Statistical analyses were performed using SPSS software (version 22.0). A *P* value < 0.05 by Fisher’s Exact test and a *p* value < 0.0167 by Kruskal-Wallis H test were considered statistically significant. All probabilities are two-tailed.

## Results

### Clinical data

**Fig. 1:**
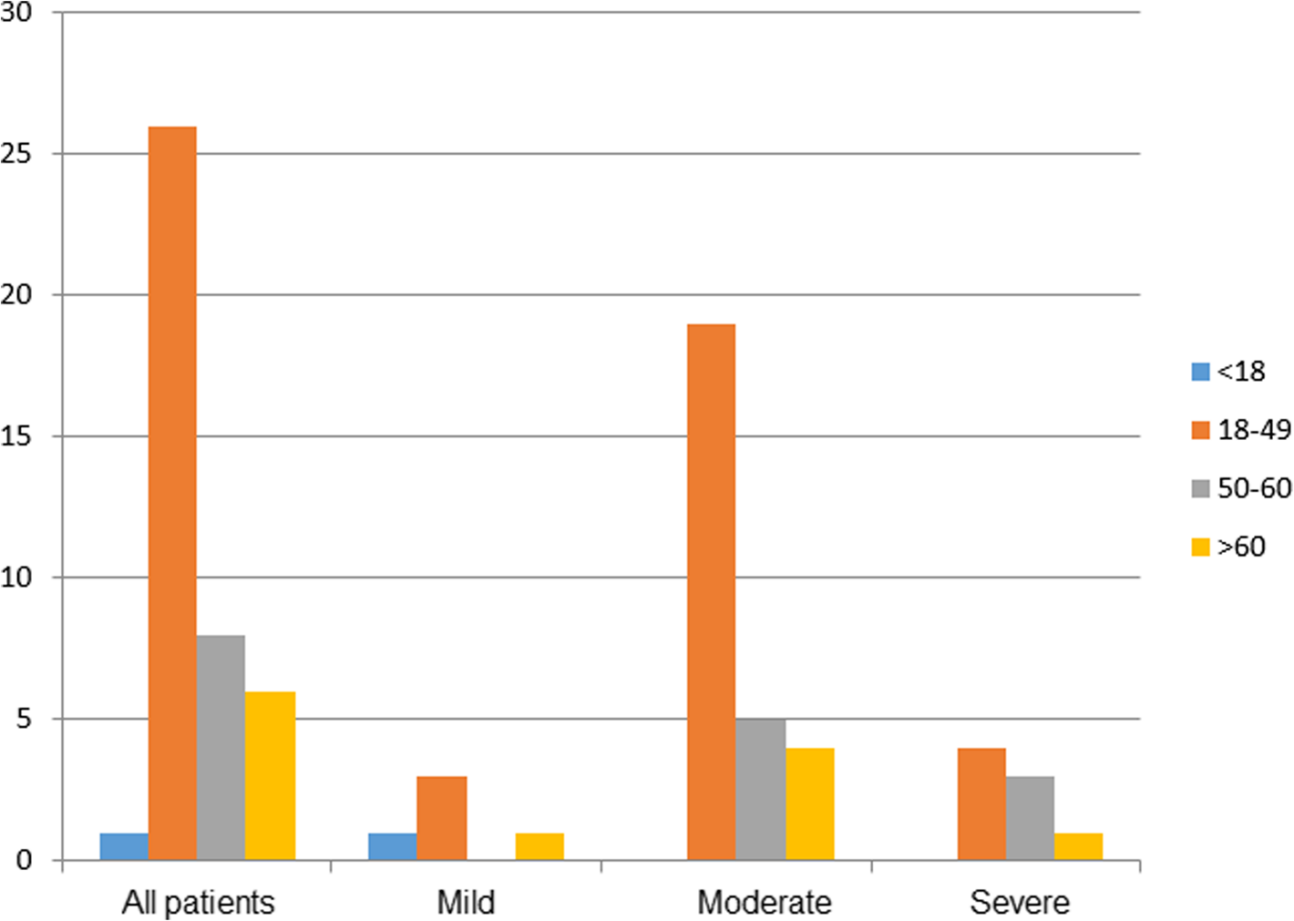
The age distribution of 41 patients diagnosed with COVID-19 by age group and the age distribution in each group

A total of 41 diagnosed COVID-19 cases were enrolled in this study. The overall median age of the 41 patients was 45 years (interquartile range, 31–53; range, 10–87 y) (Table 1, Fig. 1). The age of the 3 groups was not significantly different (Table 2). The groups with mild, moderate, or severe disease comprised 5, 28, and 8 patients, respectively. No patients entered the Intensive Care Unit, and none died. None had HBV/HCV infection. There were only one child in the mild disease group and no adolescent patients in the overall study population. The gender ratio of the 3 groups was not significantly different (Table 2). 32(78%) of the patients had received oxygen therapy (Table 1). Only 4 patients among the 3 groups (9.8%) had visited Wuhan. All of these patients were in the severe group and had received oxygen therapy, Overall, the oxygen therapy of the 3 groups was not significantly different (Table 2).

**Table 1:** Demographics and clinical signs at hospital admission in patients overall^a^

Variables	*N* = 41	
Female, (%)	18 (43.9)	
Age,(years)	45 (31,53)	
Temperature,(°C)	37.2 (36.6,38.0)	
Oxygen saturation, (%)	97 (95,99)	
Travel/Contact history, (%)		
Wuhan	4 (9.8)	
Wuhan-related	28 (68.3)	
Beijing	4 (9.8)	
Thailand	5 (12.2)	
Underlying Diseases		
Hypertension, (%)	7 (17.1)	
Diabetes, (%)	3 (7.3)	
COPD, (%)	2 (4.9)	
Coronary heart disease, (%)	1 (2.4)	
HBV/HCV Infection	0	
Laboratory results		Normal range
Lymphocyte percentage, (%)	23.91 ± 9.90	20–40
Lymphocyte count,(10^9/L)	1.29 ± 0.63	1.1–1.32
CRP,(mg/L)	11.3 (2.45,35.2)	0–6
LDH,(U/L)	226.15 ± 59.67	109–245
CK,(U/L)	78.0 (56.5138.0)	26–174
CK-MB,(U/L)	17.0 (12.0,24.5)	0–24
cTnI,(ng/mL)	1.5 (0.8,5.0)	0–0.5
Myoglobin,(mg/mL)	26.0 (19.7118.6)	0–85
AST,(U/L)	26.0 (21.0,34.0)	8–40
ALT,(U/L)	27.0 (19.3,46.5)	5–40
WBC,(10^9/L)	5.42 ± 1.73	3.5–9.5
ECG, (%)		
Normal	9 (22)	
Abnormal	31 (78)	
Lung imagings, (%)		
Nonpneumonia	5 (12.2)	
Pneumonia	36 (87.8)	
Bilateral GGO & Consolidation &Pleural effusion Unilateral GGO & Consolidation	27 (65.8)9 (22)	
Group, (%)		
Mild	5 (12.2)	
Moderate	28 (68.3)	
Severe	8 (19.5)	
Oxygen Therapy, (%)	32 (78)	
The numbers of “day of illness” at admission,(days)	4.29 ± 1.4	

^a^Reported as means and standard deviations or medians and interquartile ranges for continuous variables, and reported as number and percentage for categorical variables

*ALT* alanine aminotransferase; *AST* aspartate aminotransferase; *CK* creatine kinase; *CK-MB* creatine kinase-myocardial band; *CRP* c-reactive protein; *cTnI* cardiac troponin-I; *LDH* lactate dehydrogenase;*WBC* white blood cell count; *ECG* electrocardiograph; HBV, hepatitis B Virus; HCV, hepatitis C Virus;COPD, chronic obstructive pulmonary disease

**Table 2:** Demographics of patients with COVID-19 at hospital admission in overall population and by disease severity^a^

Variables	Mild(*n* = 5)	Moderate(*n* = 28)	Severe(*n* = 8)	P value
Gender, (%)				0.632
Male	4 (80)	15 (53.6)	4 (50)	
Female	1 (20)	13 (46.3)	4 (50)	
Age,(years)	29 (25,80)	45.5 (10,79)	47 (21,87)	0.564
Temperature,(°C)	37.5 ± 0.47	37.3 ± 1.03	37.3 ± 1.04	0.954
Oxygen saturation, (%)	97 (96.5,98)	96 (92.5,98.8)	98.5 (95.3,99)	0.403
Travel/Contact history, (%)	5 (100)	25 (89.3)	7 (87.5)	1
Underlying				
Diseases	0	5 (17.9)	2 (25)	0.564
Hypertension, (%)	0	1 (0.4)	2 (25)	0.127
1Diabetes, (%)	1 (20)	1 (0.4)	0	0.266
COPD, (%) Coronary heart disease, (%)	0	0	0	
HBV/HCV Infection	0	0	0	
The numbers of “day of illness” at admission,(days)	4 ± 1.3	4.5 ± 1	4.2 ± 1.1	0.671
Oxygen Therapy,(%)	3 (60)	21 (75)	8 (100)	0.201

^a^Reported as means and standard deviations or medians and interquartile ranges for continuous variables, and reported as number and percentage for categorical variables. *P* values across the mild, moderate, and severe disease groups were derived by Fisher’s exact test

*HBV* hepatitis B Virus; *HCV* hepatitis C Virus; *COPD* chronic obstructive pulmonary disease

Overall, fever (78%) and cough (70.7%) were the most common symptoms at onset (Table 3). Less common symptoms were sputum production (29.3%), chest tightness (29.3%), shortness of breath (26.8%), runny nose (14.6%), sore throat (14.6%), diarrhea (12.2%), and nasal congestion (9.8%). There was no significant difference in clinical symptoms among the three groups (*P* > 0.05).

**Table 3:** Cardinal symptoms of patients with COVID-19 at hospital admission in overall population and by disease severity^a^

	Patients	Mild	Moderate	Severe	P
Subjects, n	41	5	28	8	–
Fever	32 (78)	2 (40)	22 (78.6)	8 (100)	0.123
Cough	29 (70.7)	2 (40)	19 (67.9)	8 (100)	0.058
Sputum production	9 (29.3)	0	6 (21.4)	3 (37.5)	0.281
Chest tightness	9 (29.3)	1 (20)	6 (21.4)	3 (37.5)	0.628
Shortness of breath	8 (26.8)	0	5 (17.9)	3 (37.5)	0.233
Headache	4 (12.2)	0	4 (14.3)	0	0.357
Runny nose	6 (14.6)	1 (20)	4 (14.3)	1 (12.5)	0.929
Diarrhea	5 (12.2)	0	5 (17.9)	0	0.358
Sore throat	6 (14.6)	1 (20)	3 (10.7)	2 (25)	0.245
Nausea	2 (4.9)	0	2 (7.1)	0	0.614
Fatigue	3 (7.3)	0	3 (10.7)	0	0.472
Stuffy nose	3 (7.3)	1 (20)	2 (7.1)	0	0.403
Body pain	2 (4.9)	0	2 (7.1)	0	0.614
Joint pain	1 (2.4)	0	1 (3.6)	0	0.789

^a^ Reported as n (%), unless indicated otherwise. P values across the mild, moderate, and severe disease groups were derived by Fisher’s exact test

### Routine blood results

For the overall population, the routine blood tests showed that 18 (43.9%), 15 (36.6%), and 6 (12.2%) of the 41 patients had, respectively, low lymphocyte count, low lymphocyte percentage, and low white blood cell (WBC) count (Table 4). We found that the low lymphocyte count and low white blood cell (WBC) count were not statistically significant among the three groups by the Kruskal-Wallis H test and Bonferroni correction(*p* > 0.0167) (Table 5).

**Table 4:** Blood routine results of patients with COVID-19 at hospital admission in overall population and by disease severity^a^

		Patients	Mild	Moderate	Severe	P
Subjects, n		41	5	28	8	–
WBC (3.5–9.5 × 10^9^/L)	Normal	36 (87.8)	5 (100)	26 (92.9)	5 (62.5)	0.046
	Low	5 (12.2)	0	2 (7.1)	3 (37.5)	
Lymphocyte percentage (20–40%)	Normal	26 (63.4)	3 (60)	18 (64.3)	5 (62.5)	0.398
	Low	15 (36.6)	2 (40)	10 (35.7)	3 (37.5)	
Lymphocyte count (1.1–3.2 × 10^9^/L)	Normal	23 (56.1)	5 (100)	17 (60.7)	1 (12.5)	0.006
	Low	18 (43.9)	0	11 (39.3)	7 (87.5)	
CRP (0–6 mg/L)	Normal	19 (46.3)	5 (100)	14 (50)	0	0.002
	Elevated	22 (53.7)	0	14 (0)	8 (100)	
LDH (109–24 U/L)	Normal	32 (78)	5 (100)	24 (87.5)	3 (62.5)	0.007
	Elevated	9 (22)	0	4 (14.3)	5 (62.5)	
CK (26–174 U/L)	Normal	35 (85.4)	5 (100)	25 (89.3)	5 (62.5)	0.103
	Elevated	6 (14.6)	0	3 (10.7)	3 (37.5)	
CK-MB (0–24 U/L)	Normal	33 (80.5)	4 (80)	23 (82.1)	6 (75)	0.903
	Elevated	8 (19.5)	1 (20)	5 (17.9)	2 (25)	
CTnI (0–0.5 ng/mL)	Normal	0	0	0	0	0.621
	Elevated	41 (100)	5 (100)	28 (100)	8 (100)	
Myoglobin (0–85 mg/mL)	Normal	30 (73.2)	5 (100)	23 (82.1)	2 (25)	0.002
	Elevated	11 (26.8)	0	5 (17.9)	6 (75)	
AST (8–40 U/L)	Normal	35 (85.4)	5 (100)	23 (82.1)	7 (87.5)	0.571
	Elevated	6 (14.6)	0	5 (17.9)	1 (12.5)	
ALT (5–40 U/L)	Normal	27 (65.9)	5 (100)	19 (67.9)	3 (37.5)	0.064
	Elevated	14 (34.1)		9 (32.1)	5 (62.5)	

^a^ Reported as n (%), unless indicated otherwise. P values across the mild, moderate, and severe disease groups were derived by Fisher’s exact test. All blood routine results were obtained upon admission

*ALT* alanine aminotransferase; *AST* aspartate aminotransferase; *CK* creatine kinase; *CK-MB* creatine kinase-myocardial band; *CRP* c-reactive protein; *cTnI* cardiac troponin-I; *LDH* lactate dehydrogenase; *WBC* white blood cell count

**Table 5:** Blood routine results of patients with COVID-19 at hospital admission *by disease severity*^a^

Variables	Normal range	Mild(*n* = 5)	Moderate(*n* = 28)	Severe(*n* = 8)	P value
WBC,(10^9/L)	3.5–9.5	6.46 (4.18,7.86)	5.56 (4.85,6.55)	4.60 (2.97,5.03)	0.063
Lymphocyte percentage, (%)	20–40	23.6 (18.1,44.5)	21.8 (14.6,28.9)	24.4 (14.7,28.7)	0.670
Lymphocyte count, (10^9/L)	1.1–3.2	2.15 (1.14,2.43)	1.20 (0.81,1.65)	0.91 (0.65,1.18)	0.052
CRP,(mg/L)	0–6	2.10 (1.45,6.45)	7.00 (2.58,31.90)	52.45 (20.53,81.78)	0.003
LDH,(U/L)	109–245	210.0 (182.5283.0)	205.5 (181.3244.3)	258.5 (209.5311.0)	0.184
CK,(U/L)	26–174	114.0 (63.0,135.0)	75.0 (58.0,123.8)	113.0 (49.3249.8)	0.866
CK-MB,(U/L)	0–24	20.0 (16.0,64.0)	15.0 (11.0,20.0)	20.5 (12.0,56.5)	0.133
cTnI,(ng/mL)	0–0.5	1.20 (0.75,1.75)	1.30 (0.65,1.90)	9.05 (1.95,10.83)	0.007
myoglobin,(mg/mL)	0–85	22.8 (19.1,25.9)	26.0 (21.3,32.2)	141.6 (19.4171.3)	0.267
AST,(U/L)	8–40	28.0 (23.5,30.0)	25.0 (20.0,32.0)	31.5 (24.3,39.0)	0.384
ALT,(U/L)	5–40	24.0 (15.0,45.0)	29.0 (19.0,49.0)	26.5 (20.0,47.5)	0.858

^a^ Reported as median, and interquartile range (IQR) values. P values across the mild, moderate, and severe disease groups were derived by Kruskal-Wallis H test and Bonferroni correction. All blood routine results were obtained upon admission

*ALT* alanine aminotransferase; *AST* aspartate aminotransferase; *CK* creatine kinase; *CK-MB* creatine kinase-myocardial band; *CRP* C-reactive protein; *cTnI* cardiac troponin-I; *LDH* lactate dehydrogenase; *WBC* white blood cell count

CRP was elevated in 22 of the 41 patients (Table 4). Specifically, the CRP was normal for all patients in the mild group. CRP was elevated in 14 (50%) of the patients in the moderate group, and elevated in all (100%) patients of the severe group. The median CRP was 2.10 mg/L (interquartile range, 1.45–6.45 mg/L) of the mild, 7 mg/L (interquartile range, 2.58–31.9 mg/L) in the moderate, and 52.45 mg/L (interquartile range, 20.53–81.78 mg/L) in the severe (Table 5). The patients with high CRP in the severe group was significantly greater than that of the mild and moderate groups (*P* = 0.003).

Within the myocardial enzyme spectrum, cardiac troponin-I (cTnI) was elevated in all patients (Table 4). And we found that the patients in the severe group with elevated cTnI was statistical significantly higher than that of the mild and moderate groups (Table 5) (*P* = 0.007). Interstingly, we used speculated factors such as cTnI and ALT performing Pearson’s correlation analysis with LDH and CRP. The results showed that cTnI had a positive correlation with LDH (r = 0.738, *p* = 0.037) and CRP (r = 0.74, *p* = 0.042). And there was no correlation between ALT and CRP (*p* > 0.05) (Table 6). And the elevated AST and ALT had no statistical difference among three groups (*p* = 0.384 and *p* = 0.858).

**Table 6:** The ECG results of patients with COVID-19 at hospital admission by disease severity ^a^

Variable	Mild(n = 5)	Moderate(n = 28)	Severe(n = 8)	P value
ECG, (%)				0.002
Normal	1 (20)	8 (28.6)	0	
Changes of ST-T or VPC	4 (80)	11 (39.3)	0	
Flatness and inversion of T waves or Left anterior branch block	0	6 (21.4)	3 (37.5)	
QS waves in II,III and avF leads	0	3 (10.7)	5 (62.5)	

^a^ Reported as n (%), unless indicated otherwise. P values across the mild, moderate, and severe disease groups were derived by Fisher’s exact Test

*ECG* electrocardiograph

*VPC* ventricular premature contraction

### Electrocardiograph and imaging findings

**Fig. 2:**
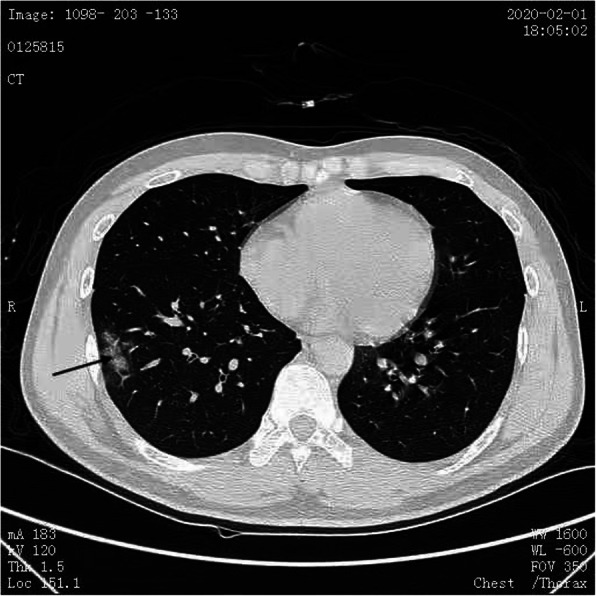
A flaky GGO was observed under the right lung pleura (black arrow)

**Fig. 3:**
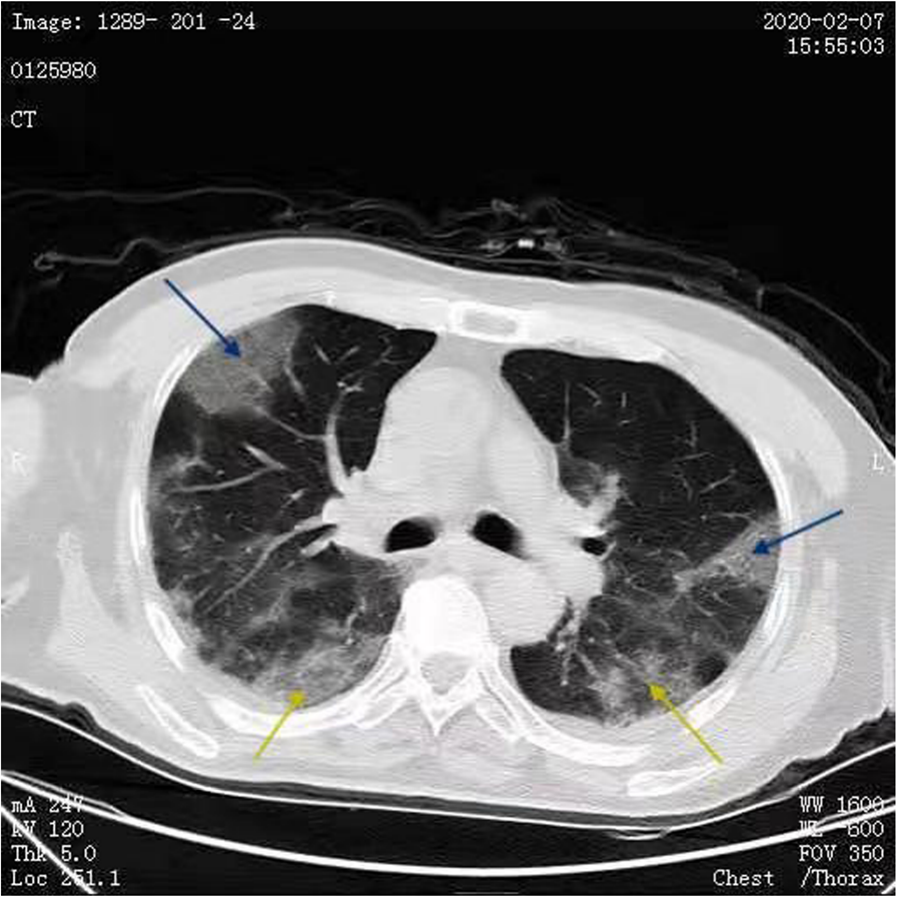
Multiple areas of patchy GGO and solid shadow (blue arrow) were seen under the pleura of both lungs with bilateral pleural effusion (yellow arrow)

Among the 41 patients overall, 32(78%) were abnormal in electrocardiograph (ECG) (Table 7). In the three groups, the changes in ECG were statistically significant (*p* = 0.002). 5(12.2%) were nonpneumonia and 27(65.8%) had bilateral distributed pneumonia (Table 8). Twenty-six patients (63.4% Fig. 2) had ground-glass opacity (GGO) shadows or had GGO combined with solid shadows (consolidation with GGO) or pleural effusion (Fig. 3). All patients in the mild group were normal on CT scan. In the moderate group, 18 patients had GGO or GGO with consolidation. (64.3%; Fig. 2). In the severe group, lesions were bilateral in all (100%) of the patients, and 8(100%) had GGO or GGO with consolidation (Fig. 3). Bilateral lung distribution and GGO or GGO with consolidation were meaningful in the three groups(*P* > 0.05).

**Table 7:** Lung CT results of patients with COVID-19 at hospital admission in overall population and by disease severity^a^

Subjects, n	Mild(n = 5)	Moderate(n = 28)	Severe(n = 8)	P value
Distribution				< 0.001
Nonpneumonia	5 (100)	0	0	
Bilateral	0	19 (67.9)	8 (100)	
Unilateral	0	9 (32.1)	0	
Characteristics				0.001
No GGO	5 (100)	10 (35.7)	0	
Consolidation & GGO & Pleural effusion	0	18 (64.3)	8 (100)	

^a^ Reported as n (%), unless indicated otherwise. P values across the mild, moderate, and severe disease groups were derived by Fisher’s exact test

*GGO* ground-glass opacity

**Table 8:** Correlation with LDH and CRP in severe group^a^

Predictive factors	LDH(U/L)		CRP (mg/L)	
	Correlation coefficient	P value	Correlation coefficient	P value
cTnI (ng/mL)	0.738	0.037	0.704	0.035
ALT(U/L)	0.321	0.678	0.397	0.331

^a^ The correlation coefficient and p value across the severe were derived by Pearson’s correlation analysis

*CRP* C-reactive protein; *LDH* lactate dehydrogenase; *cTnI* cardiac troponin-I; *ALT* alanine aminotransferase

## Discussion

This report describes the clinical characteristics of 41 patients with diagnosed COVID-19 in Changchun, Jilin Province. All the patients had a history of Wuhan-related exposure or travel. And eight of them had familial cluster disease. The median age of the patients was 45 years (interquartile range, 31, 53; range, 10–87 y). The population was stratified as mild, moderate, and severe disease based on the COVID-19 Diagnosis and Treatment Plan (Trial Version 7) announced by the National Health and Medical Commission of China [14]. Four of the patients came to Jilin after visiting Wuhan, and all of them had severe disease and required oxygen therapy, with blood oxygen saturation less than 93%. This cutoff is a guide for treating patients outside Wuhan. As it was important for patients with COVID-19 to have oxygen therapy, 32(78%) had oxygen therapy, which was consistent with other studies [15].

In this population, the majority of the patients (28, 68.3%) had moderate disease. As of 25 January, there were no adolescents among our first 41 patients, and only 1 (2.4%) child. This is consistent with other research [1]. The common history of these patients of close contact or travel is also consistent with other studies [16]. The main clinical symptoms overall and in each group were fever and cough. Few presented with significant upper respiratory signs and symptoms (e.g., nasal congestion, sneezing, or sore throat).

The pathogenic mechanism of COVID-19 has not been fully elucidated. Coronary viral receptor cells may be located in the lower respiratory tract [1, 16]. Based on the similarity of SARS-CoV-2 with SARS-CoV, it has been the fact that these viruses share the same receptor, angiotensin-converting enzyme 2 (ACE2). SARS-CoV-2 enters cells by binding to the receptor ACE2 driven by S protein; and its affinity is 10- to 20-fold that of SARS-CoV. This indicates that ACE2 has a vital role in the viral invasion of cells. Indeed, all cells that can secrete ACE2 may become target cells and are more susceptible to SARS-CoV-2 infection [17–19]. ACE2 is distributed in the heart, lung, gastrointestinal tract, and liver. Some studies have suggested that SARS-CoV-2 attack cells that secrete ACE2 in alveolar epithelial cells. This interferes with the balance of the ACE2/Ang- [1–7]/Mas axis, which leads to a series of inflammatory reactions and then respiratory discomfort such as fever, cough, and sputum production [20, 21]. Similarly, there are reports that the gastrointestinal discomfort such as nausea and diarrhea experienced by patients with moderate disease may be related to an abundance of ACE2 in digestive tract cells [22, 23].

In this study, although there were low lymphocyte counts and high LDH changes, they were not statistically significant in the three groups. This was contrary to other studies [24–28]. Due to the limited number of samples, further studies were needed to confirm the results. The elevated CRP was related to disease severity, which was in line with other studies [26–29].

In the present study, no abnormal changes were noted in the lung CT scans of the patients with mild disease. For patients in the moderate and severe groups, the most common CT features were GGO and GGO combined with consolidation. The differences were significant among the 3 groups (*P* < 0.001), which were consistent with other studies [1, 7, 16].

In our study, it was found that the elevation of cardiac troponin I (cTnI) and the changes of ECG were statistically significant in the three groups. Related literature [30–32] reported that the elevated troponin of COVID-19 patients who died was much higher than that of survivors and that cardiac troponin I was directly related to mortality. Also it was proposed that the increase in troponin may be related to heart damage or myocarditis caused by COVID-19. The related document [33] has studied that the sensitivity of myocardial cells to SARS-CoV-2 is one of the mechanisms of myocardial injury, and other mechanisms have also been studied in other relevant literatures [32, 34–36]. So far the specific mechanism of the heart damage and elevated cTnI remains unknown. According to the upper limit of troponin I [TnI] elevation greater than 99%, it is defined as heart injury. Combining the elevation of cTnI and the changes in electrocardiogram (ECG) in this study, the existence of cardiac injury is considered, which is consistent with other studies [30–32, 34–36].

In order to further clarify whether elevated troponin could also be used as a predictor for early identification of severe COVID-19, we used Pearson’s correlation analysis to analyze the correlation between cardiac troponin I (cTnI) and CRP and LDH. ***Interestingly,*** We found that cTnI was strongly positively correlated with CRP (r = 0.704, *p* = 0.042) and LDH (r = 0.738, *p* = 0.037), and related literature [26–29] had reported that both LDH and CRP can be used as powerful predictors for early identification of patients with severe COVID-19. Therefore, elevated cTnI could be considered as a candidate predictor of severe COVID-19, reflecting the prognosis of patients with severe COVID-19.

## Conclusion

In this study, the clinical characteristics of 41 confirmed COVID-19 patients in Jilin Province were retrospectively analyzed. Elevated cTnI could be considered as a predictor of severe COVID-19, reflecting the prognosis of patients with severe COVID-19. The result warrant further confirmation. As of this writing, there is no effective medicine for the treatment of COVID-19. This study may provide guidance for the treatment of patients based on clinical characteristics, a scientific basis for the screening of patients, and references for future research.

## Data Availability

The datasets generated and analyzed during the present study are available from the corresponding author on reasonable request.

## Abbreviations

GGO: Ground-glass opacity
WHO: World Health Organization
SARS-CoV-2: Severe acute respiratory syndrome coronavirus 2
CT: Computed tomography
WBC: White blood cell
cTnI: cardiac troponin-I
CK: Creatine kinase
CK: Myocardial band or CK-MB
LDH: Lactate dehydrogenase
ALT: Alanine aminotransferase
AST: Aspartate aminotransferase
ACE2: Angiotensin-converting enzyme 2
CRP: C-reactive protein
ECG: Electrocardiograph
HBV: Hepatitis B Virus
HCV: Hepatitis C Virus
VPC: Ventricular premature contraction
